# Artificial intelligence anxiety and academic burnout among Chinese postgraduate students: perceived stress and rumination as serial mediators

**DOI:** 10.3389/fpsyg.2026.1943409

**Published:** 2026-09-22

**Authors:** Maosen Long, Lina Xiong, Junhua Li

**Affiliations:** 1 Student Affairs Office, Guilin University of Electronic Technology, Guilin, Guangxi, China; 2 School of Mathematics and Computational Science, Guilin University of Electronic Technology, Guilin, Guangxi, China; 3 Communist Youth League Committee, Guilin University of Electronic Technology, Guilin, Guangxi, China

**Keywords:** academic burnout, artificial intelligence anxiety, perceived stress, postgraduate students, rumination, serial mediation

## Abstract

**Introduction:**

This study examined whether perceived stress and rumination help explain why postgraduate students with higher artificial intelligence (AI) anxiety also report greater academic burnout.

**Methods:**

We analysed data from a cross-sectional online questionnaire survey conducted among postgraduate students in Guangxi, China. The final sample included 1,501 postgraduate students. Participants completed measures of AI anxiety, academic burnout, perceived stress, and rumination. The four measurement models were examined in Mplus using weighted least squares mean and variance adjusted estimation, and serial indirect associations were tested with PROCESS Model 6 using 5,000 bootstrap samples.

**Results:**

Higher AI anxiety was associated with higher academic burnout, higher perceived stress, and stronger rumination. Perceived stress and rumination each showed significant indirect associations between AI anxiety and burnout, and the serial path through perceived stress and then rumination was also significant. The pattern remained after adjustment for sex, age, academic stage, discipline, prior AI-tool use, and weekly AI-tool use duration.

**Discussion:**

These findings indicate partial serial mediation and should be interpreted as cross-sectional indirect associations rather than evidence of temporal causality.

## 1 Introduction

Academic burnout remains a substantial concern in higher education, particularly when demands are prolonged and recovery resources are limited. In student research, burnout is typically described in terms of emotional exhaustion, cynicism toward academic work, and reduced academic efficacy (Schaufeli et al., 2002a). The construct draws on the broader burnout literature, which treats burnout as a response to chronic demands and depleted resources (Maslach et al., 2001), and it has been widely used across cultural settings as an indicator of maladaptive academic functioning (Schaufeli et al., 2002b).

Postgraduate students are an especially relevant group in which to study burnout. Their academic progress depends not only on coursework but also on research output, supervisor feedback, publication pressure, and longer-term career planning. Burnout among master’s and doctoral students was already a visible concern during the COVID-19 period (Andrade et al., 2023), and longitudinal work has linked academic burnout with lower resilience and poorer life satisfaction among medical students (Wang Q. et al., 2022). In Chinese higher education, related work has connected burnout with learning self-efficacy, teacher emotional support, intolerance of uncertainty, and self-regulatory fatigue (Yang et al., 2022; Qiang et al., 2024).

The postgraduate context adds a further layer of complexity. Research training is closely tied to identity formation, supervisor-mediated evaluation, and expectations about future academic or professional competence. Supervisor-postgraduate relationships have been linked to perceived stress through self-efficacy and psychological resilience (Liu et al., 2024), which suggests that postgraduate stress is shaped by both personal and relational resources. In this group, burnout is unlikely to reflect simple tiredness after study alone. It may also reflect uncertainty about research competence, institutional expectations, and future value in a changing knowledge economy.

The rapid integration of AI into higher education has introduced a new source of uncertainty for postgraduate students. AI tools are now used for literature searching, translation, coding, writing support, language polishing, and even early-stage research design. These uses can save time, but they also raise questions about competence, authorship, academic integrity, research norms, and future employability. AI anxiety refers to apprehension that arises from learning, using, or anticipating the consequences of AI technologies (Wang and Wang, 2022). In the original Artificial Intelligence Anxiety Scale, this anxiety includes concerns about learning AI, job replacement, sociotechnical blindness, and AI configuration (Wang and Wang, 2022).

AI anxiety should not be reduced to simple dislike of technology. Students may use AI tools frequently while still worrying that those tools are changing academic standards or diminishing the value of human expertise. This distinction matters in postgraduate education, where students may be expected to use AI efficiently while also protecting originality, judgment, and research integrity. Recent work on generative AI acceptance likewise treats AI anxiety as a psychological factor that can coexist with AI literacy and attitudes toward AI (Cengiz and Peker, 2025).

Most current studies of AI anxiety have focused on technology acceptance, AI literacy, learning motivation, and AI-related self-efficacy. Research has examined AI anxiety alongside generative AI acceptance, attitudes toward AI, AI literacy, and generative AI adoption intentions among university students (Cengiz and Peker, 2025; Hu, 2026), awareness of AI among teacher candidates (Darancik et al., 2025), and AI literacy, self-efficacy, and academic resilience in nursing education (Chen et al., 2026). Other student studies have linked AI anxiety with learning motivation and AI learning behaviour (Cagande et al., 2026; Wang Y. M. et al., 2022).

Taken together, this literature shows that AI anxiety matters for learning and technology use, but it says less about whether AI anxiety is linked to academic burnout. Some studies suggest that AI anxiety is tied to career concerns and digital well-being (AL-Nasa’h et al., 2026), which implies that its consequences may extend beyond immediate technology use. Other work has examined how AI anxiety shapes experiences of ChatGPT-supported learning and foreign language enjoyment (Gündüz et al., 2026). What remains less clear, especially for postgraduate students, is whether this anxiety is associated with broader academic strain rather than with technology attitudes alone.

We therefore expected AI anxiety to be positively associated with academic burnout. Conservation of Resources theory (COR) argues that people try to protect valued resources and experience stress when those resources are threatened, lost, or difficult to replenish (Hobfoll, 1989). Later work emphasizes that resource loss can accumulate and create cycles of vulnerability (Hobfoll, 2001). In postgraduate education, AI anxiety may signal perceived threats to time, competence, academic identity, and future career resources. This reasoning is also compatible with the Job Demands-Resources model (JD-R), which explains burnout as a consequence of high demands and insufficient resources (Demerouti et al., 2001).

AI-related pressure is not limited to one academic task. A postgraduate student may need to learn new tools, judge the reliability of AI-generated information, avoid ethical mistakes, and maintain confidence in human expertise at the same time. These demands draw on attention and self-regulatory capacity. When they persist, they may reduce the resources needed for research persistence and academic engagement. This follows the COR view that burnout can develop through cumulative resource loss rather than through isolated stressors (Hobfoll, 2001).

The school demands-resources model (Salmela-Aro and Upadyaya, 2014) helps translate the broader JD-R logic into educational settings. Academic demands can promote student burnout when students lack sufficient resources to manage them. AI anxiety may increase perceived academic demand because students must monitor technological change, learn unfamiliar tools, and compare their competence with rapidly shifting standards. If they believe that AI will weaken their academic value or make their skills obsolete, they may invest extra psychological effort to regain control. Over time, that effort may contribute to emotional exhaustion and cynicism, two central components of academic burnout (Schaufeli et al., 2002a).

Perceived stress may be one mechanism linking AI anxiety with burnout. Perceived stress refers to the extent to which people view their circumstances as unpredictable, uncontrollable, and overloaded (Cohen et al., 1983). The stress-buffering literature likewise emphasizes that psychological and social resources shape how stressors are appraised and managed (Cohen and Wills, 1985). In an AI-rich academic environment, students who feel anxious about AI may be more likely to appraise changes in research and learning practices as difficult to control. That appraisal may be especially salient in postgraduate education, where academic identity, productivity, and future career opportunities are closely connected.

This emphasis on appraisal is central to the model. Two students may face the same AI policy, use the same writing tool, or encounter the same expectation to use AI in research, yet experience different levels of stress. In PSS terms, the difference lies in whether those demands are experienced as manageable or as exceeding available coping resources (Cohen et al., 1983). AI anxiety may therefore be associated with burnout not only because AI creates objective demands, but also because students interpret those demands as uncertain and difficult to control.

The Transactional Model of Stress and Coping (TMSC; Lazarus and Folkman, 1984) further supports placing perceived stress before rumination in the hypothesized sequence. In this model, stress depends on how individuals appraise environmental demands in relation to their coping resources. AI anxiety may first shape postgraduate students’ appraisal of AI-related academic demands as unpredictable, uncontrollable, or excessive. When this stress appraisal persists, students may be more likely to repeatedly focus on the threat, its causes, and its possible consequences, thereby showing stronger rumination.

Student research already points to the importance of perceived stress in both academic and psychological outcomes. Perceived stress has been strongly associated with mental well-being among university students (Slimmen et al., 2022), and gender differences in perceived stress and coping suggest that stress appraisal is central to student adaptation (Graves et al., 2021). Among Chinese college students, perceived stress has been associated with internet addiction through procrastination-related mechanisms (Gong et al., 2021). Among postgraduate students, supervisor-postgraduate relationships have also been linked to perceived stress through self-efficacy and psychological resilience (Liu et al., 2024).

Perceived stress is also closely tied to academic burnout. Academic stress has predicted academic burnout in adolescent and college samples (Gao, 2023), and longitudinal evidence indicates that stress and burnout may be linked through indirect pathways (Zhang et al., 2025). Recent educational psychology research has also connected effort-reward imbalance with academic burnout through frustration and perceived stress (Ma et al., 2026). Intervention studies further suggest that reducing academic stress can reduce burnout among university students (Chen and Qi, 2025).

Rumination may provide a second pathway linking AI anxiety with burnout. Response Styles Theory defines rumination as repeated attention to negative mood, its causes, and its consequences (Nolen-Hoeksema, 1991). This response style can prolong distress and interfere with adaptive problem solving, and later work clarified that rumination is not simply repeated thinking but a form of repetitive negative cognition that can maintain depression, anxiety, and impaired functioning (Nolen-Hoeksema et al., 2008). The Ruminative Responses Scale (RRS) has been widely used to assess this tendency, including in Chinese samples (Han and Yang, 2009; Int-Veen et al., 2024).

AI-related concerns are especially likely to invite repetitive thinking because many of them do not have immediate closure. A student can learn to use an AI tool and still remain uncertain about whether it will alter publication standards, supervisor expectations, or future labour-market competition. Response styles theory would predict that repeated attention to such unresolved concerns prolongs distress rather than solving the problem (Nolen-Hoeksema et al., 2008). In this sense, rumination may be one cognitive process through which AI anxiety remains active during daily academic work.

AI anxiety may activate rumination through several concrete concerns. Students who worry about mastering AI tools may repeatedly compare their competence with technological change. Those concerned about job replacement may repeatedly imagine future career disadvantage. Students who are unsure about academic integrity rules may repeatedly question whether AI use will be judged as legitimate. Such thoughts can consume cognitive resources and reduce effective engagement with academic tasks, which is consistent with response styles theory (Nolen-Hoeksema et al., 2008).

Empirical studies also support the relevance of rumination to student stress and maladaptive outcomes. Perceived stress and mobile phone addiction among college students have been linked through rumination-related pathways (Peng et al., 2022). Childhood trauma and depressive symptoms among Chinese college students have likewise been examined through the combined roles of rumination and perceived stress (Wang et al., 2024). Among university students with a tendency toward generalized anxiety disorder, metacognition, rumination, and sleep have been shown to be closely related (Yu et al., 2024). Social support research during lockdowns has similarly identified rumination as a process linking social conditions with negative emotions (Guo et al., 2022).

Perceived stress and rumination may operate as an ordered process rather than as two unrelated pathways. In line with TMSC, stress appraisal can direct attention toward a source of threat, and rumination can then sustain attention to that threat. Digital mental health intervention research suggests that perceived stress and rumination can change together and may both be relevant intervention targets (Boucher et al., 2024). Research on cognitive avoidance also indicates that stress may be involved in pathways leading to rumination among college students (An et al., 2025). These findings support a model in which AI anxiety is associated with higher perceived stress, perceived stress is associated with stronger rumination, and rumination is associated with higher academic burnout. At the same time, the reverse cognitive-perseveration sequence is theoretically plausible and should be evaluated in sensitivity analyses because the present cross-sectional design cannot establish temporal precedence.

A serial indirect-effect model is useful because a direct association alone cannot show whether appraisal, repetitive cognition, or both are involved. The proposed chain model separates these processes while keeping them theoretically connected. In that respect, it follows recent educational psychology work that explains academic outcomes through linked psychological processes rather than through isolated predictors.

Recent educational psychology research increasingly uses mechanism-oriented models to explain why academic outcomes emerge. AI perception has been linked to academic engagement through academic motivation and academic self-efficacy (Shi et al., 2026a). Short video addiction has been examined in relation to academic disengagement through moderated mediation analysis (Shi et al., 2026b), and boredom proneness has been linked to disengagement through academic self-efficacy and self-control (Shi et al., 2026c). A similar logic appears in work on crisis perception, interpersonal stress, and emotional labor among teachers (Shi et al., 2024).

Against this background, we examined a serial indirect-effect model involving AI anxiety, perceived stress, rumination, and academic burnout among Chinese postgraduate students. The study asked whether AI anxiety was associated with burnout and whether this association was linked to stress appraisal and repetitive negative cognition. Because the data were cross-sectional, the hypotheses refer to theoretically specified indirect associations rather than demonstrated temporal causality. We proposed four hypotheses: H1, AI anxiety is positively associated with academic burnout; H2, perceived stress mediates the association between AI anxiety and academic burnout; H3, rumination mediates the association between AI anxiety and academic burnout; and H4, perceived stress and rumination serially mediate the association between AI anxiety and academic burnout.

The hypothesized serial indirect-effect model is shown in Figure 1.

**Figure 1:**
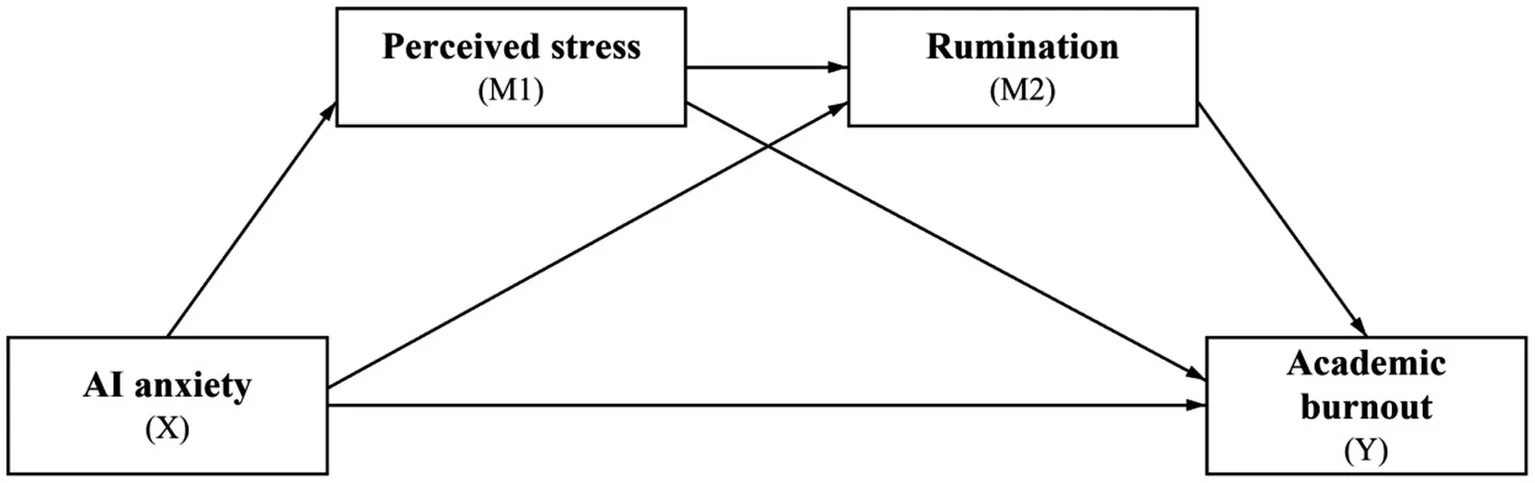
Hypothesized serial mediation model.

## 2 Materials and methods

### 2.1 Participants and procedure

We conducted a cross-sectional online survey among postgraduate students in Guangxi, China, in June 2026. Guangxi was selected as a feasible regional field setting because the research team had access to several local universities and postgraduate student communities, which facilitated recruitment of a sufficiently large sample within the study period. This choice reflects sampling feasibility rather than representativeness of all Chinese postgraduate students. Convenience sampling was used to reach eligible master’s and doctoral students. The questionnaire link was distributed electronically through university-based postgraduate communication channels, online student groups, and researcher-accessible academic networks. Participants completed the questionnaire through Wenjuanxing^1^, a widely used online survey platform in China. The final analytic dataset included 1,501 valid responses.

The sample included 774 women (51.6%) and 727 men (48.4%). Regarding age, 257 participants (17.1%) were 20–22 years old, 522 (34.8%) were 23–25 years old, 356 (23.7%) were 26–28 years old, 262 (17.5%) were 29–31 years old, and 104 (6.9%) were 32 years or older. The academic-stage distribution was as follows: 383 first-year master’s students (25.5%), 473 second-year master’s students (31.5%), 331 third-year master’s students (22.1%), 106 first-year doctoral students (7.1%), 131 second-year doctoral students (8.7%), and 77 doctoral students in the third year or above (5.1%).

In terms of discipline, 637 participants (42.4%) were from science and engineering fields, and 864 (57.6%) were from humanities and social sciences. A total of 1,429 participants (95.2%) reported prior use of AI tools, whereas 72 (4.8%) reported no AI-tool use. Regarding weekly AI-tool use, 153 participants (10.2%) reported almost no use, 201 (13.4%) reported 1–5 h, 208 (13.9%) reported 6–10 h, 494 (32.9%) reported 11–20 h, and 445 (29.6%) reported more than 20 h.

Before data collection, the opening page informed participants that the study was anonymous, voluntary, and used only for academic research. Eligible participants were postgraduate students currently enrolled in master’s or doctoral programs. Participants could withdraw at any time before submitting the questionnaire without penalty. Written or electronic informed consent was obtained before the questionnaire began.

### 2.2 Measures

AI anxiety was measured using the Artificial Intelligence Anxiety Scale (AIAS) developed by Wang and Wang (2022); later psychometric work has also examined the scale’s confirmatory structure for academic research (Alshaibani et al., 2025). The AIAS assesses anxiety related to AI learning, job replacement, sociotechnical blindness, and AI configuration. In the present dataset, 21 items were scored from 1 to 5, with higher scores indicating higher AI anxiety. All AIAS items were scored in the same direction. The questionnaire was administered in Chinese, and the item wording used in the survey was reviewed for clarity and consistency before data collection. The second-order confirmatory factor analysis showed acceptable fit, χ^2^/df = 2.465, RMSEA = 0.031, TLI = 0.997, CFI = 0.997, and SRMR = 0.024. The Cronbach’s alpha coefficient was 0.965 for the total scale, and 0.909, 0.882, 0.857, and 0.814 for the four dimensions.

Academic burnout was measured using the Maslach Burnout Inventory-Student Survey (MBI-SS). The MBI-SS was designed to assess emotional exhaustion, cynicism, and academic efficacy in student samples (Schaufeli et al., 2002a), and its factorial validity has been examined in Chinese student samples (Hu and Schaufeli, 2009). The measure is grounded in the broader burnout framework in which burnout reflects a response to chronic demands and resource depletion (Maslach et al., 2001). In the present dataset, 15 items were scored from 1 to 7. Emotional exhaustion and cynicism were positively scored, and academic efficacy items were reverse-coded when calculating the total burnout score. Higher scores therefore indicated higher academic burnout. The second-order confirmatory factor analysis showed acceptable fit, χ^2^/df = 4.153, RMSEA = 0.046, TLI = 0.998, CFI = 0.998, and SRMR = 0.021. The Cronbach’s alpha coefficient was 0.979 for the total scale, and 0.940, 0.934, and 0.948 for emotional exhaustion, cynicism, and low academic efficacy.

Perceived stress was measured using the 14-item Perceived Stress Scale (PSS) developed by Cohen et al. (1983). The PSS assesses how often individuals experience life circumstances as unpredictable, uncontrollable, and overloaded (Cohen et al., 1983), and Chinese PSS versions have been psychometrically evaluated in Chinese samples (Huang et al., 2020). Items were scored from 1 (never) to 5 (always). Items 4, 5, 6, 7, 9, 10, and 13 were reverse-coded, and higher mean scores indicated higher perceived stress. A first-order two-factor confirmatory factor analysis showed acceptable fit, χ^2^/df = 3.633, RMSEA = 0.042, TLI = 0.995, CFI = 0.996, and SRMR = 0.025. The Cronbach’s alpha coefficient was 0.949 for the total scale, and 0.904 and 0.899 for the two dimensions.

Rumination was measured using the Ruminative Responses Scale (RRS). The RRS was developed within response styles theory to assess repetitive attention to negative mood and its causes and consequences (Nolen-Hoeksema and Morrow, 1991). The Chinese version revised by Han and Yang (2009) includes 22 items and three dimensions: symptom rumination, brooding, and reflective pondering. Items were scored from 1 (never) to 4 (always), with higher scores indicating stronger rumination. The RRS was selected because the present study focused on a general repetitive negative thinking tendency that may be activated by AI-related academic uncertainty. However, because it is not an AI-specific or academic-task-specific rumination measure, this choice is also treated as a measurement limitation. The second-order confirmatory factor analysis showed acceptable fit, χ^2^/df = 2.104, RMSEA = 0.027, TLI = 0.999, CFI = 0.999, and SRMR = 0.021. The Cronbach’s alpha coefficient was 0.984 for the total scale, and 0.971, 0.934, and 0.937 for the three dimensions.

For the primary analyses, each scale was represented by its mean total score because the hypotheses concerned associations among the global constructs rather than dimension-specific pathways. This approach was consistent with the theoretical structure and psychometric evidence of the measures. Dimension-level analyses were beyond the scope of the present study.

### 2.3 Data analysis

Data were analyzed using Mplus, SPSS, and the SPSS PROCESS macro. Mplus was used to conduct CFAs of AI anxiety, academic burnout, perceived stress, and rumination. Because the indicators were ordinal Likert-type responses, the WLSMV estimator was used. Model fit was assessed using χ^2^/df, RMSEA, CFI, TLI, and SRMR (Kline, 2016). A four-factor CFA was estimated simultaneously, and convergent and discriminant validity were assessed using standardized factor loadings, composite reliability, AVE, and HTMT. Common method variance was examined using a common latent factor (CLF) sensitivity model and an unrotated Harman single-factor test (Podsakoff et al., 2003). All 72 focal indicators were declared categorical in Mplus (CATEGORICAL = all item indicators). WLSMV was selected for the specified categorical indicators; this is an estimator choice, not a claim that ordinal responses make WLSMV the only admissible estimator.

SPSS was used for descriptive statistics, reliability analyses, correlations, and regression analyses. Serial indirect effects were tested using PROCESS Model 6, with perceived stress and rumination entered as sequential mediators. Indirect effects were estimated with 5,000 bootstrap samples and 95% confidence intervals (Hayes, 2013, 2018). Given the cross-sectional design, the findings were interpreted as indirect associations rather than temporal mediation. Robustness analyses controlled for demographic and AI-use variables, assessed multicollinearity using VIFs, and tested the alternative mediator order.

## 3 Results

### 3.1 Measurement model and validity diagnostics

The combined four-construct CFA including all 72 focal items converged normally and showed good global fit, χ^2^ = 3381.930, df = 2,478, χ^2^/df = 1.365, RMSEA = 0.016, CFI = 0.993, TLI = 0.993, and SRMR = 0.022. No warnings were produced for this combined measurement model.

The standardized factor loadings ranged from 0.718 to 0.889 across the four constructs. The CR values ranged from 0.949 to 0.984, and AVE values ranged from 0.569 to 0.761. HTMT values ranged from 0.397 to 0.438, all below the commonly used 0.85/0.90 thresholds. These results supported acceptable convergent and discriminant validity for the four focal constructs.

The CLF sensitivity analyses should be interpreted cautiously. Both CLF specifications converged, but each produced a non-positive definite latent covariance matrix warning: the equal-loading CLF model involved AI anxiety, whereas the freely estimated CLF model involved rumination. These warnings indicate that the method factor was not cleanly separable from the substantive latent factors. We therefore did not treat the CLF as a definitive statistical correction for common method variance; instead, the main interpretation relies on the combined CFA, discriminant-validity diagnostics, and the limitations acknowledged below. Preliminary second-order CFA specifications were not retained for the primary analyses because of estimation warnings. No Heywood case involving negative residual variances was identified during model inspection. The combined four-construct CFA converged normally without warnings. Both CLF sensitivity models converged but yielded non-positive definite latent covariance matrix warnings; they were therefore treated as attempted but inadmissible sensitivity analyses, and adjusted structural paths are not reported.

### 3.2 Descriptive statistics and correlations

The four core variables had no missing values in the final dataset. AI anxiety had a mean of 3.720 (SD = 0.894), academic burnout had a mean of 5.407 (SD = 1.517), perceived stress had a mean of 3.726 (SD = 0.884), and rumination had a mean of 3.054 (SD = 0.842). Means, standard deviations, and correlations are shown in Table 1.

**Table 1:** Means, standard deviations, and correlations among the study variables.

Variable	M	SD	1	2	3	4
1. AI anxiety	3.720	0.894	—			
2. Academic burnout	5.407	1.517	0.387^***^	—		
3. Perceived stress	3.726	0.884	0.393^***^	0.383^***^	—	
4. Rumination	3.054	0.842	0.427^***^	0.396^***^	0.404^***^	—

^***^ *p* < 0.001.

Pearson correlations showed that AI anxiety was positively correlated with academic burnout (r = 0.387, *p* < 0.001), perceived stress (r = 0.393, *p* < 0.001), and rumination (r = 0.427, *p* < 0.001). Academic burnout was also positively correlated with perceived stress (r = 0.383, *p* < 0.001) and rumination (r = 0.396, *p* < 0.001). Perceived stress was positively correlated with rumination (r = 0.404, *p* < 0.001). These correlations provided the basic empirical conditions for testing the proposed indirect-effect model.

### 3.3 Regression analyses

Regression analyses for the primary, unadjusted serial indirect-effect model are shown in Table 2.

**Table 2:** Regression analyses for the primary serial indirect-effect model.

Model	Outcome	Predictor	B	SE	β	t	R^2^	F
M0	Academic burnout	AI anxiety	0.656	0.040	0.387	16.235^***^	0.150	263.581^***^
M1	Perceived stress	AI anxiety	0.388	0.024	0.393	16.532^***^	0.154	273.305^***^
M2	Rumination	AI anxiety	0.300	0.023	0.318	13.049^***^	0.248	247.372^***^
M2	Rumination	Perceived stress	0.266	0.023	0.279	11.442^***^	0.248	247.372^***^
M3	Academic burnout	AI anxiety	0.355	0.044	0.209	8.137^***^	0.250	165.881^***^
M3	Academic burnout	Perceived stress	0.362	0.044	0.211	8.316^***^	0.250	165.881^***^
M3	Academic burnout	Rumination	0.399	0.047	0.222	8.585^***^	0.250	165.881^***^

B = unstandardized coefficient; SE = standard error; β = standardized coefficient. ^***^ *p* < 0.001.

In the second mediator model, AI anxiety significantly predicted rumination (B = 0.300, SE = 0.023, β = 0.318, t = 13.049, *p* < 0.001), and perceived stress also significantly predicted rumination (B = 0.266, SE = 0.023, β = 0.279, t = 11.442, *p* < 0.001). This model explained 24.8% of the variance in rumination. In the final outcome model, AI anxiety (B = 0.355, SE = 0.044, β = 0.209, t = 8.137, *p* < 0.001), perceived stress (B = 0.362, SE = 0.044, β = 0.211, t = 8.316, *p* < 0.001), and rumination (B = 0.399, SE = 0.047, β = 0.222, t = 8.585, *p* < 0.001) all significantly predicted academic burnout. The final model explained 25.0% of the variance in academic burnout.

### 3.4 Serial mediation analysis

The PROCESS Model 6 results for the primary model are shown in Table 3 and Figure 2.

**Table 3:** Bootstrap indirect effects for the primary PROCESS model 6 analysis.

Indirect path	Effect	BootSE	95% BootCI	Proportion of total effect
Total indirect effect	0.302	0.030	[0.244, 0.362]	45.9%
AI anxiety → Perceived stress → Academic burnout	0.141	0.022	[0.101, 0.187]	21.4%
AI anxiety → Rumination → Academic burnout	0.120	0.020	[0.084, 0.162]	18.2%
AI anxiety → Perceived stress → Rumination → Academic burnout	0.041	0.008	[0.027, 0.058]	6.3%

Bootstrap samples = 5,000. BootCI = bootstrap 95% confidence interval.

**Figure 2:**
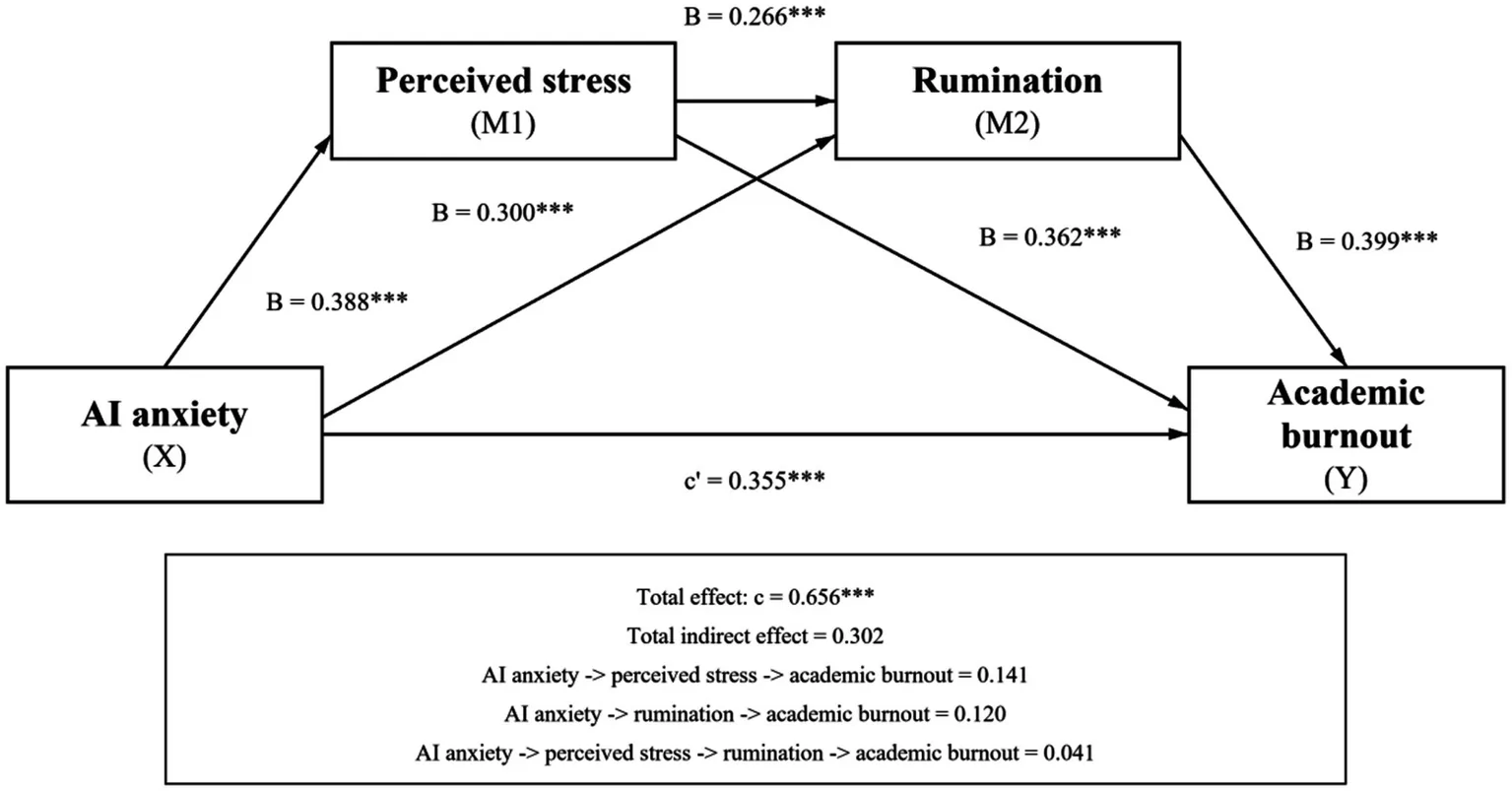
Serial mediation model linking AI anxiety to academic burnout through perceived stress and rumination. *N* = 1,501. Values are unstandardized regression coefficients. *c* = total effect; *c’* = direct effect after including the mediators. *** *p* < 0.001.

The total indirect effect was 0.302, and its 95% bootstrap confidence interval did not include zero [0.244, 0.362]. The indirect path through perceived stress was significant (effect = 0.041, BootSE = 0.022, 95% BootCI [0.101, 0.187]). The indirect path through rumination was also significant (effect = 0.120, BootSE = 0.020, 95% BootCI [0.084, 0.162]). The serial path through perceived stress and rumination was significant as well (effect = 0.041, BootSE = 0.008, 95% BootCI [0.027, 0.058]). Because the direct effect of AI anxiety on academic burnout remained significant after the mediators were entered, the findings indicate partial serial mediation rather than complete mediation. These results supported the hypothesized indirect associations in H2, H3, and H4. The component paths were B = 0.388, SE = 0.024, β = 0.393 for AI anxiety → perceived stress; B = 0.266, SE = 0.023, β = 0.279 for perceived stress → rumination; and B = 0.399, SE = 0.047, β = 0.222 for rumination → academic burnout. The direct path was B = 0.355, SE = 0.044, β = 0.209.

The covariate-adjusted model showed the same substantive pattern. After controlling for sex, age group, academic stage, discipline, prior AI-tool use, and weekly AI-tool use duration, the total indirect effect remained significant (effect = 0.301, BootSE = 0.030, 95% BC BootCI [0.244, 0.364]). The specific indirect effect through perceived stress remained significant (effect = 0.142, 95% BC BootCI [0.104, 0.194]), as did the indirect effect through rumination (effect = 0.119, 95% BC BootCI [0.083, 0.162]) and the serial path through perceived stress and rumination (effect = 0.040, 95% BC BootCI [0.027, 0.057]). A simulation-based precision check using *N* = 1,501, the observed regression coefficients and residual variances, 5,000 Monte Carlo replications, and 5,000 bootstrap draws per replication estimated 0.99 empirical detection probability for the primary serial indirect effect (0.040) and 0.99 for the reverse serial effect (0.044); median 95% interval half-widths were approximately 0.016 for both (Figure 3). This is a model-based precision assessment, not an *a priori* power analysis.

**Figure 3:**
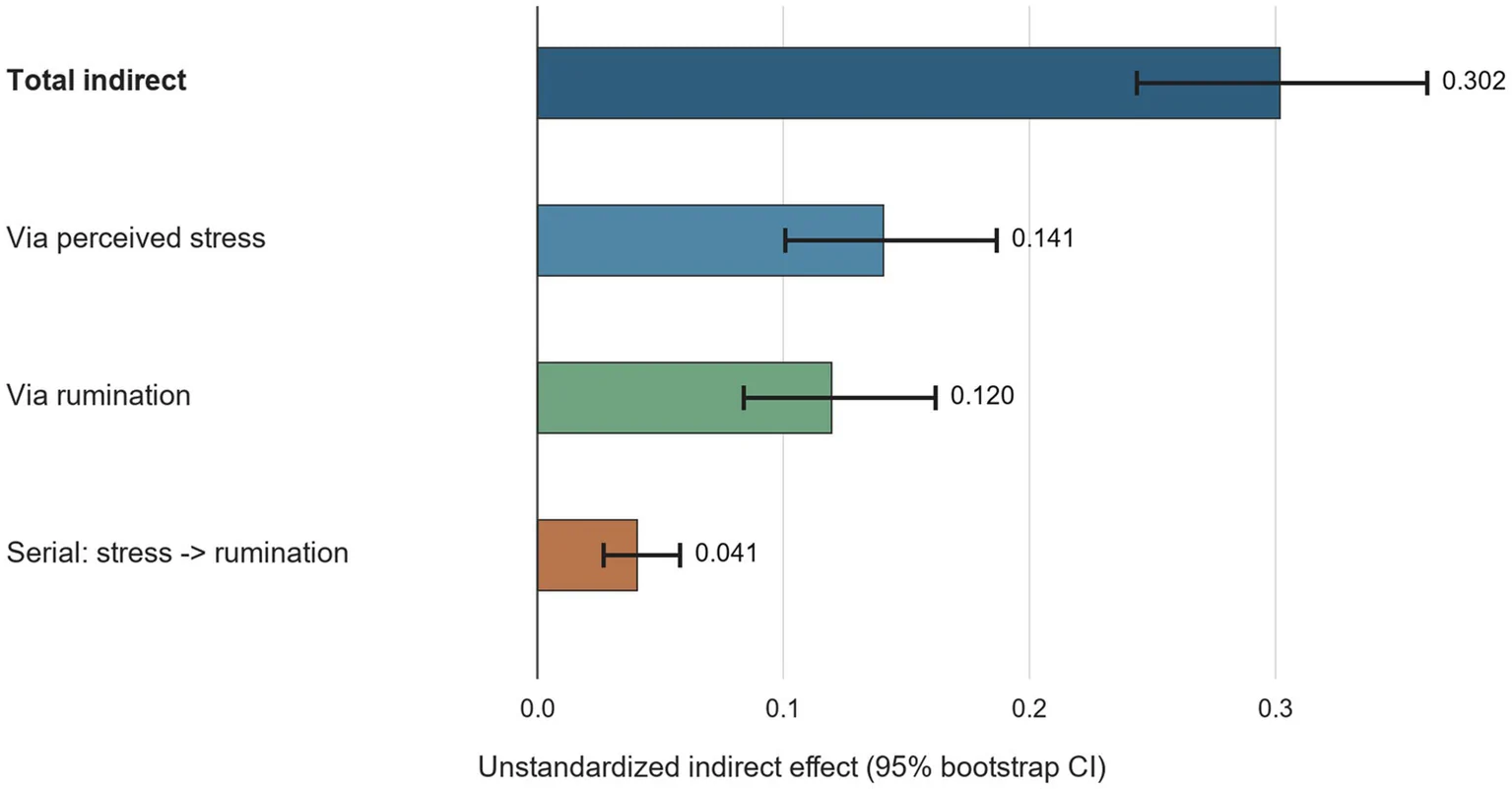
Total and specific indirect effects linking AI anxiety to academic burnout. Bars represent unstandardized indirect effects; error bars show 95% bootstrap confidence intervals based on 5,000 bootstrap samples.

### 3.5 Robustness and sensitivity analyses

The VIF diagnostics did not indicate severe multicollinearity. Using the final SAV-based diagnostic output, the maximum VIF across the three equations was 4.464 for TIME4 in the academic-burnout equation, and all values were below the commonly used threshold of 5.

The alternative serial model in which rumination preceded perceived stress was also statistically significant. In the covariate-adjusted reverse model, the serial indirect path AI anxiety → rumination → perceived stress → academic burnout was significant (effect = 0.044, BootSE = 0.008, 95% BC BootCI [0.030, 0.063]). This sensitivity finding indicates that the cross-sectional data cannot by themselves determine the temporal ordering of perceived stress and rumination. For comparison, the primary covariate-adjusted serial effect was 0.040 (BootSE = 0.008, 95% BC BootCI [0.027, 0.057]) for AI anxiety → perceived stress → rumination → academic burnout, whereas the reverse serial effect was 0.044 (BootSE = 0.008, 95% BC BootCI [0.030, 0.063]). Their similar magnitudes do not establish temporal precedence. In the reverse model, AI anxiety predicted rumination (unadjusted B = 0.403, SE = 0.022, β = 0.427; adjusted B = 0.403, SE = 0.022, β = 0.427), and rumination predicted perceived stress (unadjusted B = 0.303, SE = 0.026, β = 0.288; adjusted B = 0.298, SE = 0.027, β = 0.284).

## 4 Discussion

### 4.1 Principal findings

Three principal findings deserve emphasis. AI anxiety was positively associated with academic burnout at the zero-order level (r = 0.387) and remained a significant direct predictor after perceived stress and rumination were entered (B = 0.355). Both single-mediator paths were significant, and the serial path through perceived stress and then rumination was also significant. Because the direct path remained significant, the model should be interpreted as partial serial mediation. The same substantive pattern remained after adjustment for sex, age, academic stage, discipline, prior AI-tool use, and weekly AI-tool use duration.

In this sample, AI anxiety was not limited to attitudes toward technology use. Much of the existing literature has examined AI anxiety in relation to AI literacy, learning behaviour, or willingness to use new tools (Wang and Wang, 2022; Cengiz and Peker, 2025). Here, AI anxiety was also linked to a broader form of academic strain. That broader relevance matters because academic burnout concerns exhaustion, cynicism, and reduced academic efficacy rather than simple reluctance to use technology.

The effect was meaningful but not exhaustive. The final outcome model explained 25.0% of the variance in academic burnout, which leaves substantial variance attributable to other academic and interpersonal pressures. That is consistent with postgraduate life, where burnout may also reflect research pressure, supervisor relations, effort-reward imbalance, resilience, and institutional expectations (Al-Awad, 2024; Ma et al., 2026). AI anxiety is therefore better understood as one contributor within a broader ecology of demands.

### 4.2 Theoretical interpretation

One way to read this pattern is through resource loss. COR theory holds that stress becomes more likely when valued resources appear threatened or difficult to replenish (Hobfoll, 1989, 2001). For postgraduate students, AI may be tied to worries about competence, time, academic identity, and future employability. When keeping up with AI feels like a condition of remaining academically credible, the technology can start to look less like a neutral aid and more like another demand.

The same point appears in both the school demands-resources model and the broader JD-R model. Postgraduate students occupy a hybrid academic status: they are students, early researchers, and in many cases teaching assistants or semi-professional academic workers. AI can reduce effort for some tasks, but it can also raise expectations about speed, productivity, technical fluency, and ethical judgment (Demerouti et al., 2001; Salmela-Aro and Upadyaya, 2014). Under those conditions, the same AI environment may feel manageable to one student and draining to another. That contrast helps explain why AI exposure by itself is not enough to understand burnout; what matters is how students read the demands that come with it.

That distinction matters here because the study did not test whether AI tools are harmful in themselves. What the data capture is anxiety about AI, not simple frequency of exposure, and most participants had already used AI tools. Clear rules, adequate AI literacy, and supportive supervision may allow AI to function as a useful academic resource, whereas unclear norms and low perceived control may make the same environment feel threatening. Future work should keep exposure and anxiety analytically separate.

The perceived-stress pathway was the larger of the two single-mediator effects (effect = 0.141). This fits the idea that students who are uneasy about AI may be more likely to read academic life as unpredictable, uncontrollable, or overloaded. TMSC helps explain this mechanism: AI-related uncertainty may become psychologically consequential when students appraise it as exceeding their available coping resources (Lazarus and Folkman, 1984). In an environment where AI may affect research expectations, employment competition, and judgments about acceptable academic practice, stress appraisal provides a plausible route from AI anxiety to burnout.

That result also fits the student-burnout literature. Academic stress has been positively associated with burnout in adolescent and college samples (Gao, 2023), and longitudinal work has supported indirect links between stress and academic burnout (Zhang et al., 2025). Our findings suggest that AI anxiety may now be one more source feeding that stress process in postgraduate education.

Rumination offered a second route, and its indirect effect was also substantial (effect = 0.120). This is theoretically plausible because many AI-related concerns have no immediate resolution. Students may not know whether AI will change employment prospects, reduce the value of current skills, or alter the standards by which AI-supported work is judged. Repeated attention to those unresolved questions can consume cognitive resources that would otherwise be available for research and learning.

This mechanism is easy to imagine in routine postgraduate work. A student may finish an AI-assisted task and continue worrying about the reliability of the output, the legitimacy of the tool use, or what the tool implies about personal competence. That kind of repetitive thought does not resolve the uncertainty. In response styles theory, it becomes maladaptive when it prolongs negative affect and disrupts active problem solving (Nolen-Hoeksema et al., 2008).

The rumination result also matches broader evidence on repetitive negative thinking in student samples. Perceived stress has been linked to mobile phone addiction through the mediating role of rumination (Peng et al., 2022), and work in Chinese college students has treated rumination and perceived stress as joint mechanisms linking childhood trauma with depressive symptoms (Wang et al., 2024). The present findings suggest that AI-related academic strain may enter the same cognitive-affective channel.

The serial pathway is the most distinctive part of the model. Its effect was smaller than the two single-mediator paths (effect = 0.041), but it remained statistically reliable and followed the predicted order: AI anxiety → perceived stress → rumination → burnout. This smaller magnitude is theoretically reasonable because multi-stage psychological chains often show cumulative attenuation as an effect is transmitted across successive mediators. The finding therefore suggests that stress appraisal and repetitive negative cognition form one route linking AI anxiety with burnout, but not the only route. In this respect, the model connects COR theory, TMSC, and response styles theory within the same explanatory chain.

Even so, the ordered pathway should not be overread. It was the smallest indirect path in the model, and it supports a statistical ordering rather than a demonstrated temporal sequence. In daily life, stress and rumination may well reinforce one another more recursively than the model can show. Longitudinal and diary designs will be needed to test that process directly.

### 4.3 Practical implications

The practical implication is not simply that students need more AI training. If perceived stress and rumination help carry the AI anxiety-burnout association, students may remain vulnerable even after they learn to use AI tools competently. What may be needed is a combined response that addresses AI literacy, stress appraisal, and repetitive negative thinking together.

One practical starting point is to make expectations less ambiguous. Students need clear guidance on acceptable AI use in coursework, literature review, coding, translation, and manuscript preparation. When those boundaries are vague, ordinary tool use can become a source of perceived uncontrollability. Open discussion with supervisors may also help, particularly because supervisor-postgraduate relationships are relevant to postgraduate stress (Liu et al., 2024).

Detection tools, prohibitions, and technical workshops address only part of the problem. They may help with compliance, but they do not directly address worries about academic identity, future employability, or the changing value of human expertise. Discipline-specific guidance may therefore be especially important for postgraduate students, whose research tasks are less standardized than undergraduate coursework.

The mediation results also make stress-management and rumination-focused support hard to ignore. Mindfulness-based interventions have reduced academic stress and burnout among university students (Chen and Qi, 2025), and digital mental health interventions have reduced perceived stress and rumination among adolescents (Boucher et al., 2024). For postgraduate students, a combined program that targets AI literacy, stress appraisal, and repetitive negative thinking may be more useful than any single component alone.

Supervisors are in a particularly strong position to provide this kind of support in everyday academic work. They can help students distinguish legitimate AI-supported work from overreliance on AI, and they can encourage critical evaluation of AI outputs without turning every AI limitation into a sign of personal inadequacy. In practical terms, that kind of guidance may function as a resource that reduces uncertainty.

### 4.4 Limitations and future research

Several limitations qualify these findings. First, the cross-sectional design precludes causal or temporal conclusions. The significant reverse serial model further indicates that the ordering of perceived stress and rumination cannot be established from the present data. Longitudinal, diary, or experimental studies are needed to test temporal precedence. Second, the concurrent self-report design leaves shared method variance as a concern. Although the CLF analyses converged, their non-positive definite covariance warnings require caution. Third, convenience sampling in Guangxi limits generalizability to other regions and institutional contexts.

The measures also have limitations. AI use was assessed only by prior use and weekly duration, without distinguishing specific activities or perceived legitimacy. In addition, the general RRS may capture broad depressive cognitive tendencies rather than AI-specific or academic rumination. Future studies should use more context-specific measures and examine whether AI literacy, academic self-efficacy, supervisor support, and institutional guidance shape these associations.

Finally, the high internal consistency and strong single-scale CFA fit may partly reflect item overlap. Although the combined CFA supported convergent and discriminant validity, the measurement findings should be interpreted cautiously and replicated using multi-source or time-separated designs.

## Data Availability

The raw data supporting the conclusions of this article will be made available by the authors, without undue reservation.

## Footnotes

1
www.wjx.cn
